# Developmental Stressors Induce Innate Immune Memory in Microglia and Contribute to Disease Risk

**DOI:** 10.3390/ijms222313035

**Published:** 2021-12-02

**Authors:** Elisa Carloni, Adriana Ramos, Lindsay N. Hayes

**Affiliations:** 1 Department of Molecular and Cellular Biology, Dartmouth College, Hanover, NH 03755, USA; elisa.carloni.gr@dartmouth.edu; 2 Department of Medicine, Beth Israel Deaconess Medical Center, Harvard Medical School, Boston, MA 02115, USA; aramosam@bidmc.harvard.edu; 3 Solomon H. Snyder Department of Neuroscience, Johns Hopkins University School of Medicine, Baltimore, MD 21287, USA

**Keywords:** development, stressor, microglia, innate immune memory, training, tolerance, maternal immune activation, early life stress, ethanol

## Abstract

Many types of stressors have an impact on brain development, function, and disease susceptibility including immune stressors, psychosocial stressors, and exposure to drugs of abuse. We propose that these diverse developmental stressors may utilize a common mechanism that underlies impaired cognitive function and neurodevelopmental disorders such as schizophrenia, autism, and mood disorders that can develop in later life as a result of developmental stressors. While these stressors are directed at critical developmental windows, their impacts are long-lasting. Immune activation is a shared pathophysiology across several different developmental stressors and may thus be a targetable treatment to mitigate the later behavioral deficits. In this review, we explore different types of prenatal and perinatal stressors and their contribution to disease risk and underlying molecular mechanisms. We highlight the impact of developmental stressors on microglia biology because of their early infiltration into the brain, their critical role in brain development and function, and their long-lived status in the brain throughout life. Furthermore, we introduce innate immune memory as a potential underlying mechanism for developmental stressors’ impact on disease. Finally, we highlight the molecular and epigenetic reprogramming that is known to underlie innate immune memory and explain how similar molecular mechanisms may be at work for cells to retain a long-term perturbation after exposure to developmental stressors.

## 1. Developmental Stressors and Disease Risk

Adaptation of an internal state to a changing external environment is critical for survival and homeostasis in animals. While homeostasis is maintained by reacting to immediate stressors, allostasis is a shift in the internal state to predict and prepare for future stressors in order to deploy a more optimized response [1,2,3]. Adaptation to environmental stressors is intended to protect the animal from harm, but maladaptation can have unintended negative consequences that contribute to disease [2,4,5,6]. The concept of allostatic adaptations has been studied in metabolic syndromes, immune dysregulation, and in response to psychological and psychosocial stressors that activate the hypothalamic–pituitary–adrenal (HPA) axis [3,6,7,8]. The mechanisms of adaptation are broad and often require resetting of homeostatic set-points through negative feedback loops [1,2]. Furthermore, stressors during developmental critical periods may be particularly devastating because homeostatic set points are being established, neural circuits are solidifying, and changes in the architecture of the brain are more challenging to reverse and may thus contribute to a cascade of downstream effects [2,9,10,11]. For example, early life stress in neonatal animals led to a diminished HPA response to a subsequent adult insult long after the initial stressor [10]. In this review, we highlight the changes in brain homeostasis induced by prenatal stressors, particularly to microglia biology. We propose that alterations of microglia functions during development may be a critical mechanistic mediator of other neuronal and behavioral consequences of developmental stress. Finally, we describe the evidence for innate immune memory in microglia and their underlying molecular mechanisms. We propose that developmental stressors can lead to biological changes in microglia through similar mechanisms and may contribute to disease risk.

Development is at a sensitive period for the brain that has a greater susceptibility to environmental stressors; as a result, many types of prenatal and neonatal environmental stressors are risk factors for behavioral changes and neuropsychiatric disorders [12,13,14,15]. Extensive epidemiological studies demonstrated that prenatal immune stressors, such as infection or cytokine exposure, are linked to an increased risk for neuropsychiatric disorders including schizophrenia, autism, bipolar disorder, and attention deficit hyperactivity disorder (ADHD) [16,17,18,19,20,21,22,23,24,25]. Furthermore, higher levels of inflammation and specific intrauterine infections led to an increased risk for cerebral palsy, preterm birth, and developmental cognitive delays [26,27,28]. Prenatal exposure to psychosocial stressors, including childhood maltreatment, bereavement, family discord, unemployment, or single parenthood, increases the risk of developmental disorders of autism, ADHD, schizophrenia, depression, and cognitive delays [29,30,31,32,33,34,35,36,37,38,39]. Moreover, high prenatal levels of C-reactive protein (CRP) or cortisol led to altered amygdala connectivity and increased the risk for ADHD in boys [40,41]. Finally, prenatal exposure to drugs of abuse induces severe brain damage and behavioral and mood disorders [42,43,44]. Prenatal alcohol, opioids, amphetamines, or cocaine causes cognitive delays, attention deficits, impaired emotional development, and increased risk for other neuropsychiatric disorders such as depression and anxiety [45,46,47,48,49]. Altogether, these developmental stressors have harmful effects on the offspring with many of the stressors impacting overlapping cognitive and emotional processing in the brain. Furthermore, exposure to a combination of these environmental risk factors may have compounding impacts on brain development. For example, prenatal exposure to substance abuse is associated with a higher risk of early life adversity and could act as a two-hit model on the stress network [49,50,51]. Kirlic et al. and Lester et al. showed that children with high levels of prenatal methamphetamine or cocaine exposure and chronic postnatal stress had a blunted cortisol response (HPA activity) to acute stress in childhood, further increasing the risk for development of adult mood disorders [50,51]. With the use of animal models, the molecular impact of these developmental stressors on brain development and function is now being investigated, and we focus particularly on the microglia response to developmental stress exposure.

## 2. Developmental Stressors’ Impact on Brain Microglia

The developmental stressors highlighted above (immune, psychological, and drugs of abuse) may act through a common underlying mechanism and induce overlapping pathologies on brain development. There is evidence that immune activation occurs in all these developmental stressors, and since microglia are the primary immune cells in the brain, they may be a key mechanistic driver of disease risk. Furthermore, microglia infiltrate the brain during early embryonic development and are involved in the process of synaptic maturation and neural circuitry maintenance during development; thus, microglia are in the correct place at the right time to be impacted by the aforementioned developmental stressors. For example, several drugs of abuse can penetrate the placental barrier and act directly on the embryonic brain [43]. Once in the brain, drugs of abuse can activate microglia and astrocytes through innate immune receptors and lead to cytokine elevation in the brain [52]. Therefore, prenatal drug use could directly activate immune cells in the placenta and even microglia directly in the fetal brain, leading to a cascade of immune activation. Furthermore, early life stress can lead to concomitant immune disruption [53,54]. Acutely, early life stress leads to an elevation in stress hormones, which are sensed by microglia [55]. The long-term impact of early life stress, for example, showed that depression correlates with high cytokine expression in adolescents who previously experienced early life stress [39,56]. Therefore, immune dysregulation and potentially microglia activation could be a common pathology across developmental stressors. Thus, we explore the evidence for microglia perturbations in response to developmental stress exposure, specifically turning our attention to animal models of developmental stressors that allow for a mechanistic insight into the underlying changes to brain development, microglia biology, and testing of potential interventions.

### 2.1. Impact of Prenatal Immune Activation on Microglia

Since we hypothesize immune activation may be a common pathology of developmental stressors, we first discuss animal models for prenatal immune stress. The maternal immune-activation (MIA) animal model is generated by exposing the pregnant dam to immune activators, most commonly lipopolysaccharide (LPS), polyinosinic-polycytidylic acid (PIC), or cytokines such as interleukin-6 (IL-6), and then evaluating behavioral, neuroanatomical, and cellular changes in the offspring. MIA can induce several behavioral deficits, including impaired vocalizations, social behavior deficits, decreased prepulse inhibition, and amphetamine hypersensitivity in the offspring [17,57,58,59]. Acutely, MIA leads to high levels of cytokines and chemokines in the placenta and fetal brain [60,61]. Furthermore, several studies established that cytokines specifically in the placenta, in particular, IL-6 and IL-17A, were sufficient to drive many of the downstream behavioral deficits elicited by prenatal immune activation [61,62,63,64,65]. However, it is unclear how the early cytokine exposure mechanistically leads to the neural circuit deficits that underly the behavior or what role microglia disruption may contribute.

Several studies investigated microglia phenotypes in animals exposed to prenatal immune and identified changes in microglia density, morphology, and gene expression with highly variable results. Specifically, a systematic review of microglia phenotypes in MIA by Smolders et al. showed that microglia density was unchanged in a majority of studies, and microglia morphology was decreased in about 50% of studies [66]. Therefore, these static measures may not be the most informative metrics for evaluating microglia function and health. Mattei et al. and Hadar et al. measured inflammatory cytokine gene expression in ex-vivo microglia after MIA; however, one study showed an increase in gene expression for cytokines and immune-related molecules, including IL-6, interleukin 1 beta (IL-1β), tumor necrosis factor alpha (TNFα), CD18 (integrin beta-2), and translocator protein (Tspo), while the other showed no change [67,68,69]. These studies evaluated how prenatal immune stress alters static metrics of microglia activation, but few studies evaluated microglia functional impairments. Evaluation of microglia functional responses are important because they are more informative of microglia reactivity than static measures of morphology or density. After MIA, microglia were found to be less phagocytic [68] and motile [70]. Specifically, microglia from MIA animals showed a faster motility at an embryonic stage but then switched to a slower motility from postnatal to adulthood [54,70]. Another critical function of microglia is their immune responsiveness to exogenous stimulation. Recent studies found that MIA induced a long-term impairment in microglia responsiveness to a second immune challenge in adulthood [71,72,73]. These studies used different models (mouse vs rats) and different prenatal immune stimuli (LPS vs PIC) and were delivered at different developmental stages (between E7 and delivery); however, the results are broadly consistent, showing that microglia from MIA animals have a diminished response to a second LPS challenge in adulthood. Schaafsma et al. treated pregnant mice with LPS from embryonic day (E)15–17 then challenged the adult offspring with LPS again [71]. The microglia isolated from the whole brain of MIA animals showed a reduced gene expression for *IL-1β*, *TNFα*, and *IL-6*; in contrast, the hippocampal microglia had an enhanced cytokine expression after the second challenge, indicating MIA shows regional heterogeneity for its impact on microglia biology [71]. Clark et al. treated pregnant rats with PIC at E15 then challenged the adult offspring with LPS [72]. The MIA brain homogenates showed reduced expression of *TNFα*, *IL-6*, interferon gamma (*IFNγ*), and *IL-10* [72]. Chamera et al. treated pregnant rats with LPS from E7 to delivery (every other day), and the offspring were challenged with LPS in adulthood [73]. In brain homogenates, they found reduced expression of major histocompatibility group II (MHCII) and TNFα [73]. These microglia functions were evaluated in MIA offspring long after the prenatal stressor, which indicates a prolonged “memory” of the early life stress. An important missing gap is to identify the molecular mechanisms that are retained in microglia and that contribute to their prolonged functional impairments.

### 2.2. Impact of Early Life Stress on Microglia

Studies have demonstrated that microglia have functional receptors to sense and respond to stress hormones and neuroendocrine signals in early development [55,74], and psychological stressors during development can have long lasting effects on the brain and behavior [5,75,76]. To model human developmental stress in rodents, commonly used stressors include daily restraint and light stress to pregnant dams, maternal sleep deprivation, or maternal separation (MS) of the neonatal pups. Separation of pups from their maternal care during the early life period increased the likelihood of depression and anxiety behaviors in the offspring [77,78,79,80]. Some studies have investigated the effect of early life stress specifically on microglia by evaluating primarily microglia density and activation by either cell morphology and bulk tissue gene expression, focusing on the hippocampus and frontal cortex.

Prenatal restraint stress or sleep deprivation led to an increased microglia density and increased percentage of microglia with an activated morphology [81,82,83,84,85,86,87,88]. Some studies found this was age-specific, suggesting a change in the developmental trajectory of microglia in which microglia density was increased at postnatal day 1 but normalized by postnatal day 10 in rats [81]. Furthermore, Bittle et al. showed that direct prenatal corticosterone treatment led to increased microglia density in the offspring but not as severely as with the maternal restraint stress, indicating additional molecular mediators are simultaneously underlying the microglia phenotype [85]. However, the addition of IL-1β to the corticosterone treatment further enhanced microglia proliferation, resulting in a similar microglia density to the maternal restraint stress and suggesting that immune molecules and stress hormones may act additively in the molecular mechanism underlying early life stress [85].

In contrast to prenatal stress, studies evaluating microglia after maternal separation have had mixed results. One study showed an increase in microglia density after maternal separation [89], but another showed no change in microglia density [90]. Consistently, all the studies showed an increased number of microglia with an activated morphology [89,90,91]. To further evaluate microglia activation and function, Takatsuru et al. showed that microglia motility and process extension was increased in pups exposed to maternal separation, and the microglia motility was correlated with animal behavior, suggesting these highly motile microglia during development impact proper neural circuit maturation [91]. A couple studies treated the offspring of the early life stress with a LPS stressor to evaluate how prenatal stress impacted the functional immune reactivity of microglia [82,92]. They found that, in the hippocampus, prenatal stress led to a hyperactive LPS response with increased gene expression for proinflammatory cytokines TNFα, IL-6, and IL-18 and immune regulator NF-κB and a higher percentage of microglia with an activated morphology [82,92]. Similarly, early exposure to psychological stress also induces a long-term impaired response to subsequent HPA stressors. A meta-analysis found that maternal stress exposure caused a prolonged glucocorticoid recovery and a delayed return to baseline in the offspring after a subsequent stress challenge [93]. Furthermore, depending on the stress paradigm, the maternal separation could promote resilience to subsequent stressors, which is a prime example of effective allostatic adaptation [94]. In contrast, Pena et al. demonstrated that maternal separation led to a susceptibility to social defeat stress, which is a stress paradigm in adults [95]. A significant open question is to further evaluate the mechanisms of this resilience and at the reactivity of the microglia, specifically, in response to the secondary challenges.

### 2.3. Impact of Developmental Ethanol on Microglia

Ethanol exposure early in life can lead to an activated immune response in the mother and fetus [96]. Pascual et al. showed an elevation of IL-17 in amniotic fluid after prenatal ethanol exposure, which may activate a similar cascade described in the MIA model by Choi et al. [64,96]. Similar to the studies of prenatal psychological stress, prenatal ethanol exposure resulted in an increased microglia density, activated microglia morphology, and increased cytokine expression in the offspring brain [96,97,98,99,100,101,102,103,104]. Chastain et al. fed neonatal pups alcohol for 5 days from postnatal days 2–6, then the adult animals were challenged with LPS, and the microglia showed an increased gene expression of *IL-6* and *TNFα* and a more activated morphology [105] (Table 1). The importance of microglia and immune signaling in the etiology of prenatal ethanol exposure is growing. However, in mature animals, the microglia number and activated morphology recover after the early ethanol exposure, and the long-term functional effects to subsequent stimuli remain an open question. Furthermore, exposure to other drugs of abuse, including cocaine, methamphetamine, or opioids, have been suggested to cause neuroinflammation and microglia activation; however, these data are still controversial [106,107,108]. Moreover, how prenatal or perinatal exposure to these drugs may impact offspring microglia function is still lacking and a major outstanding question to understanding the underlying mechanism.

### 2.4. Interventions for Developmental Stress Exposures

Numerous studies have implemented various interventions to ameliorate the behavioral or anatomical phenotypes of prenatal environmental stressors. Minocycline, which inhibits microglia activation, can prevent behavioral deficits induced by maternal immune activation, maternal sleep deprivation, and neonatal alcohol exposure in rodents [67,68,87,104,105,110,111]. Deep-brain stimulation is used as a therapeutic in human patients with mood and neuropsychiatric disorders [112,113,114]. In animal models, it was found that stimulation of the medial prefrontal cortex in MIA rats prevented behavioral and neuronal deficits [69,115]. Hadar et al. showed that some brain regions such as the hippocampus and the nucleus accumbens had more microglia density following the deep-brain stimulation [69]. Peroxisome proliferator-activated receptor gamma (PPARγ) activation is another target that can be used for intervention. The pathway is known to regulate neuroinflammation. There is evidence suggesting the activation of the PPARγ pathway improves the neurogenesis and cognitive deficits in the MIA, prenatal alcohol, and maternal sleep deprivation models [88,102,116]. Similarly, bone-marrow transplants and gut-microbiota replacement have the potential to reduce psychosis symptoms in the MIA mouse model and in a human patient with schizophrenia [117,118]. N-acetylcysteine (NAC) is an antioxidant, and prenatal NAC treatment showed decreased stressed levels in pregnant dams exposed to prenatal cytokine and could be studied more as a potential therapeutic target across developmental stress models [85]. Finally, exercise also rescued the behavioral and synaptic deficits observed in MIA mice [119]. These interventions all aim to decrease proinflammatory signals and promote neuroprotective signals, which further suggests that inflammation mediated by microglia may be a common pathophysiological mediator to the stress-induced behaviors and a good therapeutic candidate.

### 2.5. Transgenerational Transmission of Developmental Stressors

To further demonstrate the long-term retention of developmental stressors, several studies showed transgenerational transmission of behavioral and neuronal phenotypes induced by developmental stressors across future generations of offspring [120,121,122,123,124]. The transgenerational transmission was found across multiple types of developmental stressors, including maternal immune activation and early life stress. These studies identified that the epigenetic reprogramming of the germ cells in both the maternal and paternal lineages was important for the transgenerational transmission of the phenotypes. This topic has been extensively reviewed elsewhere [120,123,125,126]. However, no studies have explored the transgenerational impact of developmental stressors on microglia functions to further probe the function and contribution of microglia to the ultimate disease pathology. However, recently, a study found that mice exposed to a training stimulus (described below) were able to transmit the myeloid cell phenotype across generations through epigenetic changes in the sperm [127], and these data provide a clue that the microglia memory may also be retained and transmitted across generations.

### 2.6. Summary

Altogether, these data provide compelling evidence that microglia activation may be a key mechanistic driver of the immune and “non-immune” environmental stressors; thus, it is important to evaluate if other developmental stressors may use a common mechanism to imprint on microglia and induce impaired functions. We highlighted that developmental stressors can have far-reaching impacts on microglia beyond the immediate effect of the stress exposure. An outstanding question is to determine the molecular mechanism of how microglia can remember the early life immune stress. We have a hint through the transgenerational studies that epigenetic reprogramming may be one mechanism to impart lasting changes to gene regulation on microglia; however, these ideas need to be tested more directly in microglia.

## 3. Microglia Innate Immune Memory

As described above, microglia are becoming a prime suspect in the pathology of several neurologic and neurodevelopmental disorders. Furthermore, a growing literature across neurologic disorders suggests a role for innate immune memory in modifying microglia functions as a mechanism for disease pathology. We hypothesize a similar mechanism may occur in response to developmental brain stressors. Microglia impaired by developmental insults may have altered functions and contribute to the observed phenotypes caused by developmental brain injury. An important outstanding question is to determine if young microglia can retain a memory of the developmental stressors. It is also worth asking if young microglia retain a molecular or epigenetic mark using similar mechanisms to those observed in microglia after adult immune stressors.

Innate immune memory is the systematic reprogramming of the innate immune cells after exposure to a prior immune stimulus [128]. Innate immune memory was first suggested in plants and invertebrates that lacked an adaptive immune system but showed an enhanced protective immune response after a second encounter with an infectious agent [129,130,131]. Presently, many studies have established that innate immune memory also occurs in mammals, including humans. The innate immune adaptation is divided into two categories: “immune training” leads to an enhanced immune response and “immune tolerance” leads to a suppressed subsequent immune response. An example of innate immune training is that aged mice show an enhanced response to an inflammatory challenge with LPS over younger mice, leading to a higher expression of immune molecules such as IL1β and IL6 [132]. An example of innate immune tolerance is that exposure to multiple low doses of LPS led to a serial decreased expression of immune molecules, indicating a progressive blunting of the immune response and protection against brain injury such as Alzheimer’s disease or traumatic injury [133,134]. Many molecular and mechanistic studies on innate immune memory were performed on peripheral myeloid cells (i.e., macrophages, monocytes, and natural killer cells); however, there is a growing field of studies investigating the concept of innate immune memory in microglia [135,136]. This new field has opened many new avenues of research and possibilities for therapeutic intervention in diseases.

Across several disease models, a common finding is that when the brain is in a susceptible state, the microglia immune response is often exaggerated because microglia are in a ready state to mount a rapid and enhanced immune response termed immune “priming.” For example, studies in early life alcohol exposure [105], aging [132], stroke [137], neurodegeneration [138], and prion disease [139] all showed that in the disease state, the immune response to a secondary challenge, such as LPS, led to a stronger immune response, including expression of proinflammatory cytokines TNFα, IL-1β, and IL-6. One evolutionary reason for the shift in immune responsivity is an allostatic adaptation of the microglia to be prepared for another stressor as a protective mechanism. However, numerous studies demonstrated that these hyperactive microglia can contribute to a worse neurological outcome in progressive neurodegeneration, acute brain injury, or stroke models [132,140,141,142]. In summary, several types of stressors can act as priming stimuli for microglia, and the primed microglia can be maladaptive and lead to worse disease phenotypes.

While several studies identified brain states that lead to microglia priming; there are only a few studies that established models for immune tolerance or the induction of desensitized myeloid cells that have a blunted immune response to acute activation. Chronic exposure to an immune activator, such as in sepsis, can lead to a desensitization of peripheral innate immune cells to have a reduced immune response program [143,144]. Models for microglia immune tolerance are characterized by multiple exposure to the same stressor. Therefore, multiple low doses of LPS led to a reduction in inflammatory cytokines and an increase in neuroprotective markers Arg1 and IL-10 [133,134,137,145,146,147,148]. Furthermore, this phenomenon was captured by chronic exposure to amyloid beta (Aβ). Innate immune tolerance could be a possible evolutionary adaptation to dial down the immune response program to avoid toxicity or cellular exhaustion, as in sepsis, but these adaptations can also be maladaptive. In an Alzheimer’s disease mouse model, tolerized microglia led to less accumulation of protein aggregates acutely, but a chronic repression of microglia activation in a tolerized state can also lead to an impairment of phagocytosis [134,136]. A challenge for future research is to identify clinical and timely interventions that allow pharmacological control over the microglia immune response such as treatments that can be implemented to tune up or tune down the immune response program to counter or enhance microglia reactivity.

In contrast to adaptive immune memory, innate immune memory is not antigen-specific, and many types of stimuli can cause a reprogramming of the immune response leading to susceptibility to a secondary stressor. While much of the literature to date focused on neurodegenerative stressors to prime microglia, there is emerging evidence that prenatal stressors can also induce innate immune memory. For example, Laiqi et al. looked at microglia reactivity in newborn, adult, and aged primary microglia [109,149,150]. They found neonatal microglia had a greater susceptibility of LPS-induced priming and tolerance compared with adult and aged microglia [109,149,150]. Specifically, neonatal microglia showed increased cytokine expression (TNFα, IL-6, and IL-1β) after a low-dose LPS pre-conditioning and a reduced cytokine expression after a high-dose LPS preconditioning, which was less robust in adult or aged microglia [149,150]. Furthermore, the high-dose LPS preconditioning led to an upregulation of protective cytokines (TGFβ, Arg1, IL-10, and IL-4) only in neonatal microglia [149,150]. Additionally, Ciernia et al. used a genetic autism mouse model (BTBR; a mouse strain with several autism-like behavioral phenotypes and several genetic mutations) and looked at immune response adaptation in bone-marrow-derived macrophages, highlighting specific sets of genes that were susceptible to tolerization or that showed a reduced immune response to repeated LPS exposure [151]. Specifically, *Nos1* and *Mx2* expressions were increased in the autism model compared to controls, but both also showed reduced expression after repeated LPS exposure or robust tolerization. These findings demonstrate a shift in the baseline responsiveness and in the adaptation to stress in an autism model [151]. To test this idea more directly, Schaafsma et al. treated pregnant mice with LPS in utero and then tested the immune response of the adult brain of their offspring [71]. They found a pronounced reduction in the cytokine expression in microglia with prenatal immune stress, indicating a tolerizing effect of prenatal immune stress on microglia adult function and a long-lived memory of the early life stressor [71].

Altogether, previous studies have highlighted a broad array of stressors that can induce innate immune memory in microglia (aging, stroke, prion disease, neurodegeneration, sepsis, and LPS exposure), and we highlight developmental stressors that also showed phenotypes indicative of innate immune memory (developmental alcohol exposure, maternal stress, and maternal immune activation). The priming and desensitizing adaptations are long-lasting and can be in response to cross-modal stimuli, meaning the primary stimulus and the second stimulus do not need to be the same or occur in tandem. The developmental stressors may be particularly impactful on microglia long-term function because of the susceptibility of the developmental period to reprogramming, the functional roles of microglia during development, and the long-lived lifespan of microglia *in vivo*. While studies are beginning to evaluate the retention of stressors during the developmental period, there are still many unanswered questions. (1) What types of developmental stressors can induce immune reprogramming? And are there limits on the trigger or secondary stimulus? Some studies now suggest even non-immune stimuli can induce innate immune memory, including psychosocial stress, diet, and the gut microbiome [152,153]. In addition, not many stressors have been studied as a second hit, with most studies using LPS as the immune challenge to define immune memory, but some other potential secondary stressors include neurodegeneration [134,136] and pathogens [154,155]. (2) How long does the reprogramming last? Studies about the heterologous effects of vaccines are a classic example of how a primary immune activation can have long-lasting (years) effects in circulating monocytes [156], and it has been elegantly demonstrated that the transmission of innate immunity can occur across generation [127]. However, this question has not been studied in microglia. Furthermore, the sustained effects of trained immunity in peripheral myeloid populations are known to be mediated by the direct reprogramming of bone marrow progenitor cells that explains how trained immunity can be sustained during years or even decades [157,158]. In the case of microglia, one of the most long-lived resident macrophages with a capacity for self-repopulation, the time window for memory maintenance is expanded in comparison to other myeloid populations, but we still need to better understand how microglia are able to repopulate under physiological conditions and how that process gets altered during inflammation in order to fully understand how immune memory is achieved in these cells.

In summary, we provided evidence to establish a solid foundation on which to hypothesize that prenatal stressors could act as a training or tolerizing stimuli and could impact the secondary stressors that microglia are naturally exposed to, be it phagocytosis of neuronal debris during development, normal pathogen exposures in adulthood, or degeneration later in life. As a result, trained or tolerized microglia responses may be impacted by those prenatal immune stressors and may contribute to the negative health outcomes.

## 4. Molecular Mechanism Underlying Innate Immune Memory in Microglia

Microglia are plastic cells equipped to perform immune surveillance of the brain in the same way that peripheral monocytes and other populations of resident macrophages do in their respective organs of residency, and, as previously described, they are capable of developing innate immune memory. We learned from peripheral myeloid populations that innate immune memory is metabolically and epigenetically regulated [128,159], with the mammalian target of rapamycin—hypoxia inducible factor alpha (mTOR-HIFα) axis defined as a molecular master regulator of the reprogramming [160].

From a metabolic standpoint, increased aerobic glycolysis, glutaminolysis, and cholesterol synthesis are important key pathways that mediate the training response in myeloid cells [86,161,162]. Although innate immune tolerance has not been so amply studied, we learned that the metabolic mechanisms regulating sepsis-induced and LPS-induced tolerance are mediated by fatty acid oxidation and the blockage of aerobic glycolysis [163,164]. In addition, training or tolerance can be programmed by direct modulation of enzymes from the Krebs cycle [163,165,166]. The mechanistic regulation of the training response in microglia is also mediated by aerobic glycolysis and the AKT-mTOR-HIFα axis [134,136], while tolerance was linked with the inability to induce aerobic glycolysis through the same mechanisms [134,136,167]. The contribution of metabolic pathways other than glucose metabolism have not been explored in the immune reprogramming of microglia cells. Recent studies have demonstrated that microglia are metabolically flexible cells capable of using glutaminolysis and fatty acid oxidation as sources of energy [168]; hence, it is very likely these pathways play a role in the immune reprogramming of microglia cells, overall in situations where brain glucose is scarce.

At the epigenetic level, the main four epigenetic factors linked with immune memory in myeloid cells are histone modifications, DNA methylation, chromatin modelling, and microRNAs [128]. In particular, the established histone hallmarks that accompanied trained immunity are histone 3 lysine 27 acetylation (H3K27ac), histone 3 lysine 4 methylation (H3K4me1), histone 3 lysine 4 trimethylation (H3K4me3), and low DNA methylation [128]. As far as we are aware, few studies have systematically addressed the epigenetic regulation of training and tolerance in microglia using preclinical models of aging and neurodegenerative diseases [134,169]. In these studies, H3K27ac and H3K4me1 were reportedly higher in enhancer regions from trained microglia [134,169], while a reduction in those hallmarks was found in enhancer regions from tolerant microglia [134,167,169]. In addition, early life immune stress was reported to increase DNA methylation and disrupt H3K9ac in the brain, though this was not demonstrated directly in microglia [170,171]. Brain immune tolerance caused by early life stressors were reported in similar studies, although epigenetic hallmarks that demonstrate memory to be involved in the reprogramming of this response were not shown [149,150,172]. Paradoxically, features of immune training were found in the brain of mice subjected to early life stress, with enhanced expression of IL6, IL1β, complement, and other inflammatory cytokines [67,68,111,173]. These changes in the brain of adult mice exposed to early life stress were demonstrated to rely on microglia activation [67,68,111,173].

The epigenetic and metabolic responses that regulate immune memory are tightly intertwined; metabolic rewiring modifies the epigenetic landscape, and, conversely, epigenetic mechanisms regulate the expression of metabolic genes. As part of this intertwined regulation, histones, de/acetylases, and methylases are known to be directly regulated by enzymes of the Krebs cycle, and, as previously mentioned, mTOR and HIF1α tightly control the transcriptional regulation of glucose transporter 1 and glycolytic enzymes, which are crucial for the induction of immune training [174,175]. Furthermore, it is not surprising that among the transcription factors that contribute to permissive (i.e., H3K27ac and H3K4me) or repressive (i.e., H3K27me3 and H3K9me) histone modifications and reshape the chromatin landscape to induce innate immune memory are members of the interferon regulatory factors (IRF) and signal transducer and activator of transcription (STAT) families, as well as NFκB [176,177,178,179,180,181]. For example, IFNγ, which canonically mediates its response through STAT1, can prevent the tolerization of monocytes [182] and microglia [136] by activating the mTOR–AKT–HIFα axis and boosting glycolytic metabolism, an effect that is probably concomitantly linked with an increase in permissive histone modifications.

Based on previous studies, it is not simple to clarify if a stimulus could cause training or tolerance in microglia cells (Table 2). Timing, sequence, strength, and duration of the stimuli are probably the source of what a priori could be interpreted as mixed results [135]. Two additional factors that should be considered when interpreting these data that add more complexity to the regulation of immune memory in microglia cells are: (1) microglia diversity during neurodevelopment [183] and (2) distinctive chromatin landscapes associated with microglia development [184]. Neurodevelopment is the stage in which microglia have the highest diversity [183], even without considering any regional specificity. Furthermore, the regions of the genome that are susceptible to alteration change across development based on the epigenetic marks and chromatin accessibility in maturing microglia. Therefore, this diversity should be considered when the priming stressors occur during pre- and perinatal stages. It is plausible that the same stimuli applied with the same strength, duration, and timing might distinctively affect different microglia populations based on regional and epigenetic availability.

## 5. Summary

We highlighted three types of developmental stressors that can impact microglia biology. We proposed that the diverse stressors may contribute to behavioral deficits and disease risk by utilizing common or compounding immune mechanisms. We provided evidence from animal studies that indicate microglia are capable of innate immune memory and gave several molecular mechanisms that mediate innate immune memory that have been identified in myeloid cells, including some evidence for microglia. While there are many questions that remain, we want to emphasize three key open questions. (1) What types of stimuli could impart innate immune memory in microglia? (2) What diverse conditions (timing, dose, duration, and sequence) can tip the balance between priming and desensitization? And (3) are the same molecular mechanisms found in peripheral myeloid cells also at work in microglia? These are all active areas of research and a growing field to follow in the future. As microglia become the target of many new therapeutics, it is important to keep in mind their malleability and permanence to manipulations.

## Figures and Tables

**Table 1:** Tissue and microglia (MG) phenotypes after exposure to various developmental stressors.

Model		Tissue Phenotype	Microglia Phenotype	Reactivity		Citation	
				2nd Stim	Response		
MIA	Rat, E15, PIC	↑IL-1β and TNFα (RNA) in whole Hpc	↑MG density in NAc, ↑Iba1 (protein) in the Cb and Hpc	none		[67]	Mattei 2014
MIA	Mouse, E15, PIC	↑Tspo binding and IL-6 (protein) in whole Hpc	↑Iba1 and CD18 (protein) in Cb and Hpc, ↓MG phagocytosis in Hpc	none		[68]	Mattei 2017
			MG RNA-seq: ↓genes inflammatory response, phagocytosis, and cell migration. ↑genes for synaptic plasticity, VEGF signaling, and glial cell migration				
MIA	Rat, E15, PIC		↑MG density and soma size in NAc and Hpc, ↑MHCII (protein) in Ctx	none		[69]	Hadar 2017
MIA	Mouse, E12 or E15, PIC		↑MG motility (velocity) at E18, ↓MG motility (velocity) at P10	LPS	↑MG directional motility at P42 (after E12 MIA)	[70]	Ozaki 2020
MIA	Mouse, E15–16–17, LPS			LPS	↓IL-1β, TNFα, and IL-6 (RNA) in MG from the whole brain	[71]	Schaafsma 2017
					↑IL-1β (RNA) in MG from Hpc		
					↑IL-1β, TNFα, and IL-6 (RNA) in whole Hpc		
MIA	Rat, E15, PIC	At P35: ↑TNFα ↓IL-4 and IL-10 (RNA) in whole brain		LPS at P35	↑IL-1β, ↓IL-6, IL-4 and IL-10 (RNA) in the whole brain	[72]	Clark 2019
				LPS at P60	↓IL-6, TNFα, IFNγ, and IL-10 (RNA) in the whole brain		
MIA	Rat, E7, E10, E13, E16, E19, LPS	↑CD200R (RNA), ↓CD200R (protein) in FC		LPS	mild changes in gene expression of inflammatory molecules in FC and Hpc in MIA-responsive and MIA-non-responsive mice	[73]	Chamera 2020
MIA	FIRS: Rat, E20, intra-amniotic LPS			LPS at P5	↓activated MG density, ↑IL-1β, ↓IL-6, TNFα, Cxcl10, Ccl2 (RNA) in the Hpc	[109]	Singh 2021
ELS	Rat, mild random stress, E4–20	↑IL-6 (RNA) in Hpc				[54]	Zhang 2016
ELS	Rat, maternal forced swim, E10–20		↑ramified MG density and ↓ameboid MG density	none		[81]	Gomez-Gonzalez 2010
ELS	Mouse, maternal restraint stress, E12 –E20	↑IL-1β (RNA) in Hpc	↑MG density, ↑MG activated morphology	LPS	↑IL-6, TNFα, IP10 (RNA) in the Hpc, ↑MG with activated morphology	[82]	Diz-Chavez 2012
ELS	Mouse, maternal restraint stress, E12 –E20	↑IL-1β and TNFα (RNA) in Hpc	↑MG activated morphology in Hpc	LPS	↑TNFα in the Hpc, ↑MG density in Hpc	[83]	Diz-Chavez 2013
ELS	Mouse, stress environment, E13–17	↑Aif1 and Tlr9 (RNA & protein) in Hpc	↑MG density in Hpc	none		[84]	Cohen 2016
ELS	Mouse, maternal restraint stress, E12 –E20		↑MG total density, ↑density of ameboid MG	none		[85]	Bittle 2018
ELS	Mouse, IL-1β, E12–13		↑MG total density, ↑density of ameboid MG				
ELS	Mouse, corticosterone, E12–13		↑MG total density, ↑density of ameboid MG				
ELS	Rat, 72H maternal sleep deprivation, E4, E9, or E18	↑IL-1β, TNFα, IL-6 (RNA), ↓IL-10 (RNA) in the Hpc	↑MG activated morphology	none		[86]	Zhao 2014
ELS	Rat, 72H maternal sleep deprivation, E18	↑IL-1β, TNFα, IL-6, CD68, iNOS, ↓IL-10, IL-4, Ym1, Arg1, Cd206 in the Hpc	↑MG density, ↑Iba1 (protein) in Hpc	none		[87]	Zhao 2015
ELS	Rat, 72H maternal sleep deprivation, E18	↑IL-1β, TNFα, IL-6, ↓IL-10, IL-4, Ym1, Arg1 in the Hpc	↑MG density with activated morphology	none		[88]	Han 2020
ELS	Mouse, maternal seperation, P1–21		↑MG density and MG density with activated morphology at P14, ↑MG phagocytosis at P28 in Hpc, MG RNA-seq: altered immune modulators	none		[89]	Delpech 2016
ELS	Rat, maternal separation, P1–14	↑IL-1β, TNFα in Hpc	↑MG density with activated morphology	3H maternal separation	↑IL-1β, ↓TNFα in Hpc, ↑TNFα and IL-6 in Hypo, ↑corticosterone	[90]	Roque 2016
ELS	Mouse, maternal seperation, P2–14		↑MG motility			[91]	Takasturu 2015
ELS	Mouse, 180 min maternal separation, P1–21			LPS	↑Iba1 in Hpc	[92]	Wu 2021
ELS	Mouse, 15 min maternal separation, P1–21				↓Iba1, Nlrp3, IL-18, NFκB in the Hpc		
EtOH	Mouse, 10% drink, preconception to P21	↑IL-1β, Cxcl1, MCP1, MIP1α, IL-17, CD11b, and MHCII (protein) in whole Ctx	↑Iba1 (protein) in the Ctx	none		[96]	Pascual 2017
EtOH	Mouse, 3.5g/kg EtOH, P2–9		↑MG density in the Cb	none		[97]	Kane 2011
EtOH	Mouse, 4 g/kg EtOH, P4–9	↑IL-1β and TNFα (RNA) in Ctx, Hpc, and Cb	↑MG activated morphology in the Hpc, Ctx, Cb	none		[98]	Drew 2015
EtOH	Rat, 4h EtOH vapor, P3–5	↑IL-1β and TNFα (RNA) in Cb	↑MG activated morphology in the Cb	none		[99]	Topper 2015
EtOH	Mouse, 3–5 g/kg EtOH, P7–8	↑IL-1β and TNFα (RNA) in Ctx	↑density of ameboid MG, ↑MG activated morphology, ↑Itgb2, P2ry12 (RNA), ↑CD68 (protein) in the Ctx	none		[100]	Ahlers 2015
EtOH	Mouse, 2 g/kg EtOH, E6–E18	↑TNFα, IL-12a, IL-10 (RNA), ↓IL-6 and TGFβ (RNA) in the Ctx	↑MG density, ↑MG activated morphology in the Ctx	none		[102]	Komada 2017
EtOH	Mouse, 2.5 g/kg EtOH, P5	↑MCP1 and IL-6 (protein) in the spinal cord	↑Iba1, CD68, and P2×7 (protein) in MG in the spinal cord	none		[103]	Ren 2019
EtOH	Rat, 2.5 g/kg EtOH, P2–6	↑TNFα, MCP1, Csf1r, and TLR4 (RNA) in Hypo	↑MG density, ↑MG activated morphology, ↑Iba1 protein intensity in the Hypo	none		[104]	Shrivastava 2017
EtOH	Rat, 2.5 mg/kg EtOH, P2–6	↑TNFα, IL-6, Csf1r, and TLR4 (RNA) in Hypo	↑activated MG density in the Hypo	LPS	↑IL-6 and TNFα (RNA) in MG, ↑activated MG density in the Hypo	[105]	Chastain 2019

**Table 2:** Molecular mechanisms and metabolic interventions underlying innate immune memory in monocytes and microglia.

Cell-Type	Model	Immune Paradigm	1st Stim	Reactivity		Metabolic Intervention	Citation	
				2nd Stim	Response			
**Monocytes**	Human primary monocytes	Training	b-glucan	N/A	↑ TNFα, IL-6, HIFα and mTOR pathway, ↑ glycolysis and H3K27Ac hallmark in relevant promoter regions	Training is blocked with metformin and wortmanin	[160]	Cheng 2014
**Monocytes**	Human primary monocytes	Training	b-glucan or fumarate	LPS	↑ TNFα and IL-6, ↑ glycolysis, glutaminolysis and cholesterol synthesis	Training is blocked by metformin	[161]	Arts 2016
**Monocytes**		Tolerance	LPS	LPS	↓ glycolysis, glutamynolysis, and cholesterol synthesis			
**Monocytes**	Human primary monocytes	Training	b-glucan	LPS	↑ TNFα, ↑ glycolysis, cholesterol synthesis pathway, and TCA cycle, ↑ H3K27Ac hallamrk in relevant promoter regions	Training is blocked by fluvastatin (through cholesterol synthesis pathway)	[162]	Bekkering 2018
**Monocytes**		Training	Mevalonate	LPS	↑ TNFα, ↑ glycolysis and TCA cycle, ↑ H3K27Ac hallamark in relevant promoter regions			
**Monocytes**	Human primary monocytes from patients with IgD syndrome	Training	Accumulation of mevalonate caused by mutations in mevalonate kinase	LPS	↑ TNFα, IL1β, and IL6, ↑ glycolysis and mTOR pathway			
**Monocytes**	Human BMDMs	Tolerance	LPS	LPS	↓ TNFα, IL1β, IL12, and IL6	Tolerance is blocked in the absence of Glutamine and a-ketoglutarate	[163]	Liu 2017
**Monocytes**	Human monocytes from sepsis patients	Tolerance	Sepsis	LPS	Trancriptomic data determined ↓ of ample array of inflammatory interleukines and chemokines. Although their expression is ↑ at basal levels (w/o LPS stimulation)		[164]	Shalova 2015
**Monocytes**	PBMCs from chronic mucocutaneous candidiais patients	Impaired Training	STAT1 mutation	b-glucan, LPS	Equal TNFα and IL-6 as stimulated control		[176]	Ifrim 2015
**Monocytes**	PBMCs from hyper- immunoglobulinaemia E syndrome	Training	STAT3 mutation	b-glucan, LPS	↑ TNFα, IL6			
**Monocytes**	Human primary monocytes from LPS exposed human	Tolerance	LPS	LPS	Epigenetic and transcriptomic data determined ↓ signatures of permissive hallmarks		[179]	Novakovick 2016
**Monocytes**	Human primary monocytes from LPS exposed human	Impaired Tolerance	LPS	b-glucan+LPS	Epigenetic and transcriptomic data determined ↑ signatures of permissive marks			
**Monocytes**	Human primary monocytes	Training	b-glucan	LPS	Epigenetic and transcriptomic data determined ↑ signatures of permissive marks			
**Monocytes**	Human primary monocytes	Tolerance	LPS	LPS	Epigenetic and transcriptomic data determined ↓ signatures of permissive hallmarks			
**Monocytes**	PBMCs from patients with sepsis	Tolerance	Sepsis	LPS	↓ TNFα, IL1β, and IL-6, ↓ glycolysis	IFNγ (rescued tolerance)	[182]	Cheng 2016
**Microglia**	APP23 mice	Training	1×LPS	AD model/ Ab amyloid accumulation	↓ IL-10 in brain, ↓ accumulation of Aβ, Epigenetic and transcriptomic data determined an involvement of HIFα, and ↑ signature of permissive marks		[134]	Wendeln 2018
**Microglia**		Tolerance	4×LPS	AD model/ Ab amyloid accumulation	↓ IL-1β in brain, ↑ accumulation of Ab, Epigenetic and transcriptomic data determined ↓ signature of permissive marks			
**Microglia**	Stroke mouse model	Training	1×LPS	Ischemia	↑ IL-1β, ↓ IL-10 in brain, Epigenetic and transcriptomic data determined an involvement of HIFa and ↑ signature of permissive marks			
**Microglia**		Tolerance	4×LPS	Ischemia	↓ IL-1β in brain			
**Microglia**	Mouse primary microglia culture	Training	1×Ab		↑ IL-1β, ↑ glycolysis and ↓ TCA		[136]	Baik 2019
**Microglia**	Mouse Primary microglia culture	Tolerance	3×Ab		↓ IL-1β, ↓ glycolysis			
**Microglia**	5×FAD mice	Tolerance	Endogenous Ab aggregates	Aβ iv injection	↓ IL-1β, TNFα, and CCL2 among others, ↓ motility			
**Microglia**	5×FAD mice	Impaired Tolerance		Intervention with IFNγ	↑ TNFα, ↑ glycolysis, ↑ phagocytic capacity	IFNγ (rescued tolerance)		
**Microglia**	Mouse primary microglia	Training	Low dose LPS	LPS	↑ TNFα and IL-6		[149]	Lajqi 2019
**Microglia**		Training	b-dectin	LPS	↑ TNFα and IL-6			
**Microglia**		Tolerance	High dose LPS	LPS	↓ TNFα and IL-6			
**Microglia**	P6–P12 mouse pups	Training	Low dose LPS	LPS	↑ TNFα, IL-6, IL-1β, iNOS		[150]	Lajqi 2020
**Microglia**	Adult mice	Training	Low dose LPS	LPS	↑ TNFα, IL-6, IL-1β, iNOS			
**Microglia**	Aging mice	Ablated Training	Low dose LPS	LPS	Equal TNFα, IL-6, IL-1β, iNOs as in stimulated control			
**Microglia**	P6–P12 mouse pups	Tolerance	High dose LPS	LPS	↓ TNFα, IL-6, IL-1β, iNOS			
**Microglia**	Adult mice	Tolerance	High dose LPS	LPS	↓ TNFα, IL-6, IL-1β, iNOS			
**Microglia**	Aging mice	Impaired Tolerance	High dose LPS	LPS	Equal TNFα, IL-6, IL-1β, iNOS as in stimulated control			
**Microglia**	Mouse primary microglia culture	Tolerance	LPS	LPS	↓ IL-1β, TNFα, IL-6	Tolerance is Relb mediated	[167]	Schaafsma 2015
**Microglia**	Wild type mice	Tolerance	LPS	LPS	↓ IL-1β, TNFα, IL-6	Tolerance is Relb mediated		
**Microglia**	Wild type mice	Tolerance	LPS	LPS	Epigenetic and transcriptomic data determined ↓ signatures of permissive marks		[169]	Zhang 2021
**Microglia**	Ercc1 KO mice	Training	Aging	LPS	Epigenetic and transcriptomic data determined ↑ signatures of permissive marks			

## Abbreviations

MG	microglia
Stim	Stimulus
MIA	maternal immune activation
PIC	polyinosinic:polycytidylic acid
Hpc	Hippocampus
Cb	cerebellum
Ctx	cortex
LPS	lipopolysaccharide
FC	frontal cortex
Hypo	hypothalamus
P	postnatal day
E	embryonic day
FIRS	fetal inflammatory response syndrome
ELS	early life stress
H	hour
EtOH	ethanol
BMDM	bone marrow derived macrophages
PBMCs	peripheral blood mononuclear cells
APP23	amyloid precursor protein
5xFAD	5 familiar Alzheimer’s disease mutation mouse model
Ercc1	excision repair cross-complementation group 1
A	amyloid beta
